# Characterizing PCDH19 in human induced pluripotent stem cells (iPSCs) and iPSC-derived developing neurons: emerging role of a protein involved in controlling polarity during neurogenesis

**DOI:** 10.18632/oncotarget.5757

**Published:** 2015-09-21

**Authors:** Claudia Compagnucci, Stefania Petrini, Norimichi Higuraschi, Marina Trivisano, Nicola Specchio, Shinichi Hirose, Enrico Bertini, Alessandra Terracciano

**Affiliations:** ^1^ Unit of Neuromuscular and Neurodegenerative Diseases, Bambino Gesù Children’s Hospital, IRCCS, Rome, Italy; ^2^ Confocal Microscopy Core Facility, Research Laboratories, Bambino Gesù Children’s Hospital, IRCCS, Rome, Italy; ^3^ Central Research Institute for the Pathomechanisms of Epilepsy, Fukuoka University, Fukuoka, Japan; ^4^ Division of Neurology, Bambino Gesù Children’s Hospital, IRCCS, Rome, Italy

**Keywords:** PCDH19, human iPSCs, iPSC-derived neurons, neuronal polarity, neural rosettes, Neruoscience Section

## Abstract

PCDH19 (Protocadherin 19), a member of the cadherin superfamily, is involved in the pathogenic mechanism of an X-linked model of neurological disease. The biological function of PCHD19 in human neurons and during neurogenesis is currently unknown. Therefore, we decided to use the model of the induced pluripotent stem cells (iPSCs) to characterize the location and timing of expression of PCDH19 during cortical neuronal differentiation. Our data show that PCDH19 is expressed in pluripotent cells before differentiation in a homogeneous pattern, despite its localization is often limited to one pole of the cell. During neuronal differentiation, positional information on the progenitor cells assumes an important role in acquiring polarization. The proper control of the cell orientation ensures a fine balancing between symmetric (giving rise to two progenitor sister cells) *versus* asymmetric (giving rise to one progenitor cell and one newborn neuron) division. This process results in the polar organization of the neural tube with a lumen indicating the basal part of the polarized neuronal progenitor cell; in the iPSC model the cells are organized in the ‘neural rosette’ and interestingly, PCDH19 is located at the center of the rosette, with other well-known markers of the lumen (N-cadherin and ZO-1). These data suggest that PCDH19 has a role in instructing the apico-basal polarity of the progenitor cells, thus regulating the development of a properly organized human brain.

## INTRODUCTION

The organization of functional neural circuits requires the precise and coordinated control of cell-cell interactions at nearly all stages of development, including neuronal differentiation, neuronal migration, axon outgrowth, dendrite harborization, and synapse formation and stabilization. This coordination is brought about by the concerted action of a large number of signaling factors and cell surface receptors, whose dynamic regulation enables neurons (and astrocytes) to adopt their proper roles within the developing neuronal networks. In this context, neuronal adhesion emerges as one major unexplored process, mediated by a large variety of molecules including protocadherins.

The relevance of protocadherin family in human pathology is demonstrated by many reports [1] and by the fact that human PCDH19, as member of δ-Protocadherin family is clearly involved in pathogenic mechanism of a neurological disease. In 2008 Dibbens et al. [2] reported for the first time *PCDH19* mutations in a few families in whom epilepsy and intellectual disability was restricted to females. *PCDH19* gene segregating on the X chromosome shows a peculiar X-linked inheritance, involving only heterozygote female carriers and sparing hemyzygote transmitting males. A “cellular interference” model has been invoked as the pathogenic mechanism; based on this hypothesis the coexistence of a mixed cell population (mutated/wild-type) in heterozygote females leads to a scrambled neuron-neuron communication followed by hyper-excitability. Conversely, the homogeneous neuronal population in wild-type or hemyzigote males ensures a correct synaptic connection and thus a suitable signal conduction. PCDH19 belongs to δ -Protocadherins, a major cadherin superfamily encompassing more than 80 molecules expressed primarily in the developing vertebrate nervous system and evolutionary conserved along the vertebrate lineage [3]. Based on the genomic location and structure, the protocadherin sub-family is divided in clustered protocadherin (C-PCDH)(α, β, and γ’) and non-clustered protocadherins (NC-PCDH)(δ1 and δ2). As a member of NC-PCDH, PCDH19 segregates in an isolate X-chromosome locus. Evolutionary analysis of X chromosome reveals the presence of an X and Y homologs block (comprising i.e. PCDH11 and PCDH19) with a high percentage of nucleotide (98.1%) and amino acid (98.3%) identity [4]. This evidence reflects a recent evolutive divergence of this locus on the Y Chromosome. Interestingly, the genes mapping in this Y-linked block are expressed primarily in the brain, congruous with the finding that protocadherins are predominantly expressed in the central nervous system [5]. These data are consistent with the hypothetic role of prothocadherins in the segmental development and functional differentiation of the brain throughout various species [6, 7]

Even if several Pcdh genes are recently associated to a variety of human neurodevelopmental disorders [8], the clear cellular functions, molecular mechanisms of protein interaction, and signaling partners have yet to be determined for several Pcdh subfamilies, and definitive evidence for Pcdh roles in synaptic recognition and adhesion does not yet exist.

Several studies show that d-Pcdhs can mediate Ca 2^++^ -dependent homophilic cell-cell adhesion, although binding appears to be generally weaker than that of classical cadherins. These data suggest that Pcdhs are poorly involved in typical cell-cell adhesion but might play a role in intercellular regulation.

Recently some authors described a cis-complex between N-cadherin and Pcdh19 *in vitro* and *in vivo*; they showed that disruption of this interaction impairs cell movements during neurulation in zebrafish leading to a severe alteration in early brain morphogenesis caused, at least in part, by defective cell movements in the anterior neural plate [9]. Starting from the description of PCDH19 in neurogenesis of the zebrafish model, we explore the role of PCDH19 in the development of human central nervous system. We take advantage of human induced pluripotent stem cell (iPSCs), obtained from healthy subjects, to recapitulate the role of PCDH19 in neuronal differentiation [9, 10]. The iPSC system offers the unique opportunity to explore *in vitro* neurogenesis mimicking the closure of the neural tube. The formation of typical morphological structures is comparable to those arising during embryogenesis in human physiology. Moreover, adherent culture protocols lead to the formation of Neuronal Stem Cells (NSC) arranged radially around a lumen termed “neural rosettes”. These structures exhibit functional similarities to the neural tube, producing laminar structures mimicking the layers formed during corticogenesis in the developing forebrain. This high similarity of morphogenetic and patterning events is very useful to dissect human nervous system development *in vitro* and to improve the future knowledge and management of disease model as “PCDH19 female restricted epilepsy”. Moving from the pcdh19 characterization in the zebrafish model [9], we used iPSC-derived neural rosettes to recapitulate the role of PCDH19 in human neurogenesis focusing in particular on the establishment of apical-basal polarity and the geometry of proliferation. We focused on the female iPSC model to characterize the PCDH19 biological function in order to transfer the acquired information on the *PCDH19* related epilepsy model that needs to be developed in the future.

## RESULTS AND DISCUSSION

### Geometry of proliferation

**Figure 1:**
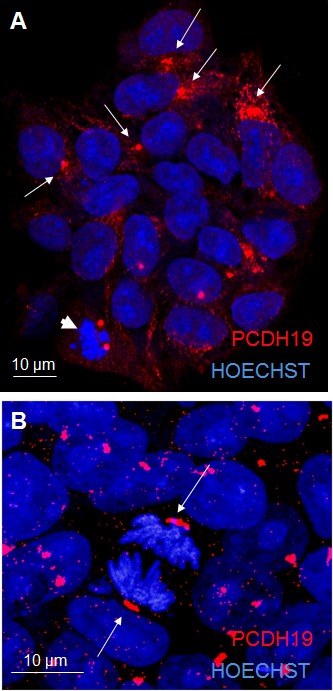
PCDH19 localization in iPSC colony The confocal images in A and B represent proliferative iPSCs with the red signal indicating PCDH19 antibody staining. The arrows in A point to the focal and polarized PCDH19 signal. The arrows in B point to the specific localization of PCDH19 at the poles of mitotic cells.

Since studies on human PCDH19 are lacking, we performed the characterization of PCDH19 subcellular localization in proliferative human iPSCs mimicking the early stage human embryo (i.e. blastocyst). Using immunofluorescence analysis for PCDH19, we demonstrate a focal localization of the signal with a higher intensity at one pole of the cell and a diffuse weaker intensity along the rim of the plasmatic membrane (Figure 1A). As expected, PCDH19 shows membrane localization typical of other adhesion molecules [11]. Interestingly, the focal localization suggests that PCDH19 might contribute to the intrinsic positional information of a pluripotent stem cell. These data demonstrate the presence of PCDH19 in proliferative iPSCs, allowing us to speculate about its expression at the early stage of human development. Further evidence of PCDH19 localization is confirmed by immunofluorescence performed on male iPSCs (data not shown).

In proliferating iPSCs undergoing cellular division, the PCDH19 signal labels the two poles of the diving cell defining the mitotic spindle (Figure 1B). The same geometry is evident in Figure 1A where a cell at earlier phase of cell division shows the same split signal at the two poles (see arrowhead in Figure 1A). Interestingly, a similar subcellular arrangement is evident for ASPM, which is critical for mitotic spindle function in mammalian cells [12–14]. Moreover, in the central nervous system ASPM emerges as a molecule regulating symmetric *versus* asymmetric cell division and ensuring the correct size of the mammalian neocortex [15]. In fact, the right mitotic spindle orientation balances between the self-renewal of the progenitor pool and the maturation of the cortical layers, and contributes to establish the correct brain size and architecture. It follows that neuronal polarized cells should particularly express proteins that maintain this spindle orientation, especially during symmetric proliferative divisions.

Mutations in ASPM are the most common cause of primary microcephaly in human, indicating a direct role for this protein in regulating cerebral cortical size [16]. While ASPM shows a clear correlation between morphological data and clinical presentation of the patients, for PCDH19, even if we find a similar cellular localization, brain development abnormalities have never been described [17], but despite this, a prenatal mild microcephaly could not be excluded due to the impossibility to perform in utero measurements. In the present study, we show a clear localization of PCDH19 at the mitotic spindle, suggesting a hypothetic role in regulating cleavage plane orientation.

### The neural rosette and the acquirement of neuronal polarity

**Figure 2:**
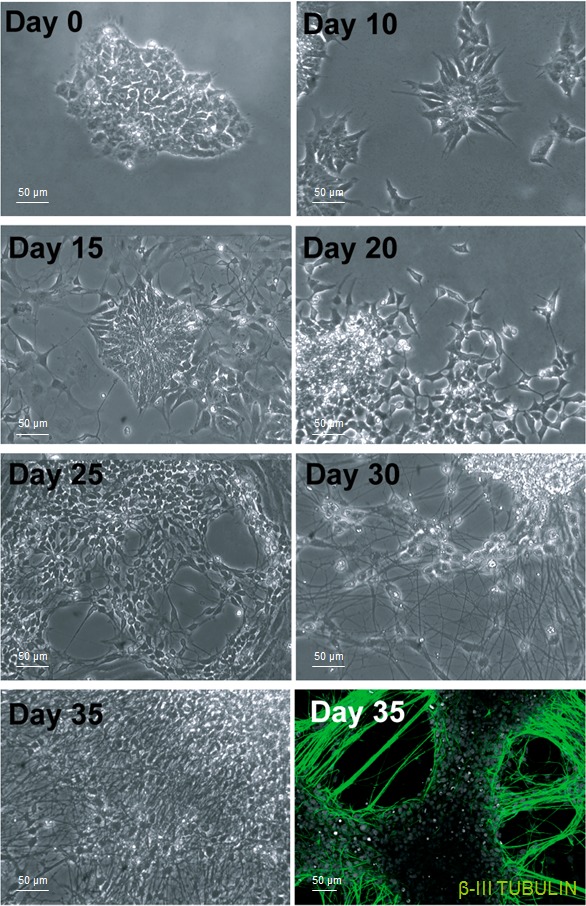
*In vitro* cortical neurogenesis Bright field images taken at different days during the differentiation of iPSCs into cortical neurons show the reorganization of the cells and their changes in cell morphology. The immunofluorecence in the bottom right corner indicate that the cells have successfully differentiated in neurons, positive to the β-III TUBULIN antibody.

Following the characterization of PCDH19 in proliferative iPSCs, we decided to focus on the placement of the cadherin adhesion molecule in differentiating neurons, in particular when the neurons acquire the first morphological polarity. For this purpose, the iPSC system allows to dissect different stages of neuronal differentiation *in vitro*. We differentiated iPSCs into neural precursor cells in monolayer using an established protocol [18]. We generated cortical neural rosettes using several growth factors at different times and observed that after 10 days the iPSCs rearranged into well-organized and polarized structures, named neural rosettes. Importantly, these structures have structural and functional similarities to the neural tube during embryogenesis, recapitulating the morphogenetic and patterning processes. This regional specificity seems to be maintained from day 10 to day 15 of *in vitro* neurogenesis (Figure 2), following this, the peculiar arrangement of the cells disappears and the neuronal precursors give rise to newborn neurons with small neurites (at day 20). In the subsequent 10 days, neurite outgrowth and harborization take place progressively. Once the neurons are mature, the neurites establish cell-cell contacts and give rise to the complex neuronal network visible in the last stage of *in vitro* neurogenesis (day 35, Figure 2).

Recent studies demonstrate that pluripotent stem cells committed to the neuronal fate are organized in neural rosettes, that are radial glial-like and have the ability to generate a variety of neural cell types. These structures have functional and morphological similarities to the embryonic neural tube. Similarly to the neural tube, the rosettes display localized zones of proliferation and can be patterned by signaling molecules and growth factors, suggesting that their formation and differentiation are governed by similar mechanisms [10, 19]. These similarities allow to study *in vitro* the morphogenetic and patterning events occurring during brain development. As already well-known, the tight junction protein zona occludens 1 (ZO-1) labels the apical side of the cells surrounding the lumen of the neural tube (despite of its even distribution in proliferating stem cells, (see Figure S1) [20] and importantly ZO-1 also maintains this lumen specific localization in the neural rosettes [10].

**Figure 3:**
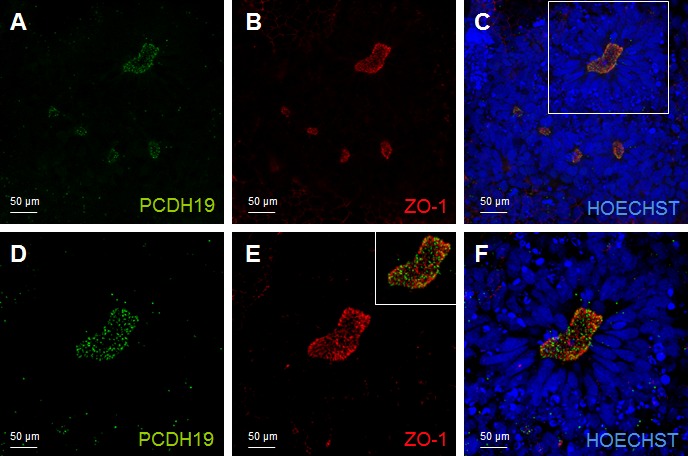
PCDH19 localization in the lumen of the neural rosette Confocal images of immunofluorescence for PCDH19 and ZO-1. The immunofluorescence for PCDH19 is localized at the lumen of the neural rosette, which is indicated by the localization of the known marker of this highly polarized structure ZO-1 **A.**, **B.**, **C.** The white box in C indicates the region magnified in **D.**, **E.**, **F.** The inset in E represents the merge image of D and E.

We took advantage from the neural rosette system to characteriz PCDH19 expression in the stage when neuronal polarity arises; using ZO-1 as a marker of neural rosette lumen, we observed that PCDH19 signal recalls the same distribution of ZO-1. The immunofluorescence analysis shows a dotted PCDH19 expression at the center of the rosette, partially overlapping with ZO-1 antibody (Figure 3).

The neural tube, as well as the neural rosettes, consists of a radially arranged neurepithelium surrounding a central lumen. The side of the epithelial cell layer facing the lumen (ventricle in the neural tube) is considered apical, while the side facing the outer surface is considered basal (Figure 3).

**Figure 4:**
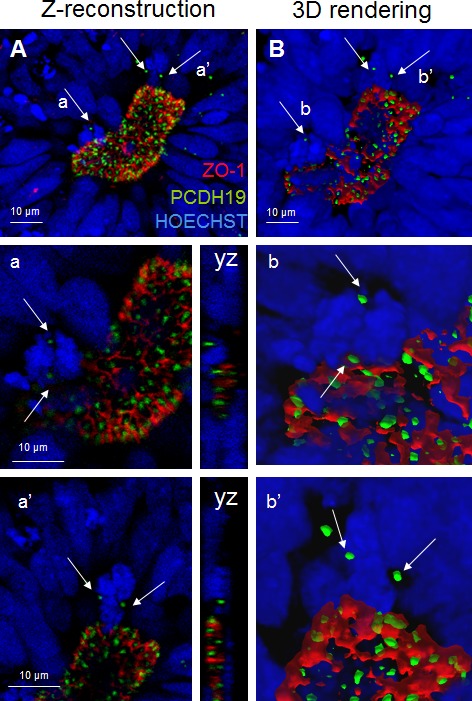
PCDH19 localization in the lumen of the neural rosettes Confocal image of the neural rosette lumen **A.** with arrows pointing to the foci of PCDH19 localization in the mitotic cells a and a’. Confocal central plane of a and a’ cells is magnified below and the yz axes are reported on the right side. **B.** 3D surface rendering of the region of interest. On the right (b and b’) 3D reconstructions of mitotic cells are reported. Bars: 10 μm.

The neural rosette, mimicking the early neural tube, appears to be organized into a stratified epithelium that is actually composed of a single layer of radial cells. These cells making contact with both apical and basal surfaces, have nuclei arranged at different levels, producing the appearance of a multi-layered structure. As indicated in Figure 3, proliferating radial cells are largely restricted to the zones which are adjacent to the lumen (center of the neural rosettes, ventricular and subventricular zone of the neural tube). During cell division, nuclei of actively dividing radial progenitors migrate toward the apical surface, where they undergo mitosis and cytokinesis (interkinetic nuclear migration) providing the apical surface of a large pool of NSCs. Importantly the PCDH19 localization at the mitotic spindle marks the cleavage plane orientation indicating symmetric (leading to proliferative) *versus* asymmetric (neurogenic) division in relation to the rosette lumen (Figures 3–4). By confocal studies we demonstrated that in proliferating pluripotent stem cells, even in the polarized cells of the neural rosette, two PCDH19 signals are localized at the level of the mitotic spindle as well, indicating that the plane of cell division occurs at the lumen. (Figure 4) These data show once again the presence of the adhesion molecules in the spindle pole orienting process, supporting the role in the positional information pattern of proliferative as well as in differentiating cells. As shown in Figure 4A different planes of spindle pole orientation are evident (cell a and b), in particular cell “a” seems to have a perpendicular spindle pole while cell “b” shows a parallel pole orientation respect to the lumen (as indicated with dashed lines in Figure 4B). However, the specific role of PCDH19 and its relation with other adhesion molecules (ZO-1, N-cadherin) need further investigations.

### PCDH19 in mature polarized neurons

**Figure 5:**
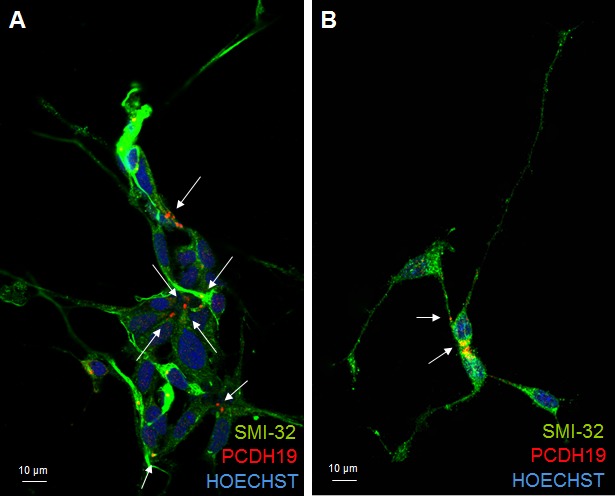
PCDH19 localization in mature neurons Confocal photographs of immunofuorescence for PCDH19 (red) and SMI32 (green) on iPSC-derived cortical neurons after 30 days of differentiation. The images in **A.** and **B.** clearly show that PCDH19 protein is present at the site of cell-cell contacts in neuronal cultures (positive to the neuronal marker SMI32) as indicated by the arrows. In particular, in A the cell-cell contact is mainly at the level of cell soma, while in B the top neuron presents the PCHD19 signal between the neurite and the cell soma of the neighboring cell.

In terminally differentiated neurons the same focused and polarized signal, observed in the other stages of neuronal differentiation, is evident for PCDH19 protein. Interestingly, PCDH19 localization occurs at the cell-cell contact between the soma of two neurons and between the distal neurite and the cell soma of the neighboring neuron (Figure 5), suggesting that it is required for the maintenance of cell-cell contact, as also shown by the juxtaposition of PCDH19 signal with ZO-1 (Figure S2). Further studies are necessary to understand the role of PCDH19 in defining neuronal networks in relation to other well-known adhesion molecules (N-cadherin), mainly expressed at the synapsis [21]. In synapses, there is a special form of adherens junction named the puncta adherentia that mediates synaptic adhesion, and this is the site where the trans interaction of cadherins occurs between pre- and postsynaptic membranes. Because of these localizations, cadherins are considered involved in various processes that are related to synaptic structures and functions.

## CONCLUSIONS

The role of cadherin family is very important in development and homeostasis of central nervous system. Although the involvement of δ -PCDHs in the pathogenesis of several human cancer types, and neural and other diseases is well established, our understanding of their molecular functions and their interplay with various signaling pathways is limited.

The recent biochemical experiments [e.g., 22, 23] that have elucidated molecular mechanisms of Pcdh interactions have actually shown that it may be more difficult than initially believed to understand the cellular and developmental processes in which these molecules participate. With this in mind, we can likely look forward to many surprises as further studies of these far -from-prototypical adhesion molecules emerge.

**Figure 6:**
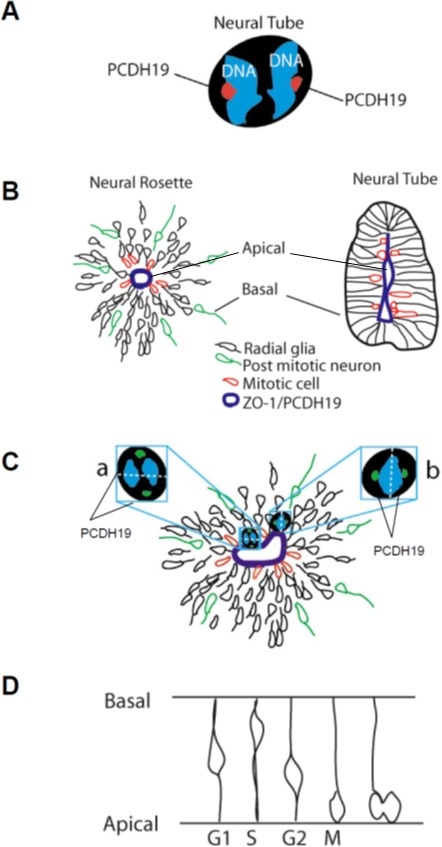
PCDH19 in proliferating and differentiating iPSCs Drawing of a dividing iPSC with PCDH19 positive foci indicated in red **A.** Schemata of a neural rosette (on the left) and of a neural tube (on the right), showing the correspondence between their lumen (positive to ZO-1) and indicating in red mitotic cells, in green post-mitotic neurons which are migrating away from the lumen and the radial glia (or progenitor cells) **B.** In **C.** a model of the rosette photographed in Fig 3 and 4 is reported to focus the attention on the cell “a” and “b”, which present different spindle pole orientations indicated by the position of the PCDH19 positive foci. The drawing in **D.** represents the interkinetic nuclear migration observed during the development of the nervous system.

Here, for the first time we characterize PCDH19 expression and subcellular distribution in human pluripotent stem cells, observing a peripheral and focal signal. We have been able to follow PCDH19 localization before, after differentiation, and at different stages of neuronal maturation. Moreover, in both proliferating and differentiating cellular types, PCDH19 marks the spindle pole during mitosis (Figure 6A, 6C).

In order to improve the knowledge on the role of PCDH19 in *in vitro* neurogenesis, we have focused on neural rosettes as a relevant tool to recapitulate brain development. In particular, our results suggest that PCDH19, labeling the center of the neural rosette (corresponding to the lumen of the neural tube, see Figure 6B, 6C), may be involved in the establishment or maintenance of the complex positional information necessary for the proper development of the human brain architecture [21]. PCDH19 localization at the spindle pole of the dividing cells, suggests that it is involved in the control of asymmetric versus symmetric cell division during *in vitro* neurogenesis. In particular, mitotic cells are evident at the center of the neural rosette (Figure 6B) and during *in vivo* neurogenesis at the apical side of the neural tube (Figure 6D). These descriptive data should be interpreted as provisional, as further work is needed to clarify the specific role of PCDH19 in human neurogenesis. It will, thus, be important to consider carefully the results of biochemical and proteomic experiments in human systems in order to define biological partners and inter actors involved in this complex process.

## MATERIALS AND METHODS

### iPSCs

Healthy female and male iPSCs were purchased from Coriell Institute (USA, Cod GM23411, GM23338, GM23340). The iPSCs were derived from human fibroblasts and reprogrammed using the episomal or viral technology (Minicircle DNA and mc-iPS Cells, Euroclone).

Phase-contrast photographs were taken with a Motic AE21 microscope (Motic Instruments Inc.) connected to a Moticam 2300 digital camera using the software Motic Images Plus 2.0.

Cell Culture Conditions. Following thawing, iPSCs were grown on MEFs (Life Technologies) for the first 4-5 weeks and then in feeder free condition using Matrigel (BD Biosciences, San Diego) in mTeSR1 (Stemcell Technologies). When the iPSCs are 70-80% confluent, they were passaged (using EDTA treatment) 1:4 and transferred to new wells in feeder-free condition and incubated at 37°C, 5% CO_2_, 20% O_2_, the medium was changed every day and the cells split every 3 days.

### Cortical neuron differentiation

For the differentiation assays, iPSC colonies were dissociated and the cells plated at a density of 1000 cells/cm^2^ into a chemically defined medium. Neuronal differentiation has been adapted from Zeng et al. (2010). Cells were kept in a medium containing DMEM/F12 (Life Technologies), N2 Supplement (Gibco) and 2 μg/ml Heparin (Sigma) for 16 days. On the 17th day, the medium has been replaced with Neural Basal Medium (Life Technologies) supplemented with N2, B27 (Gibco), BDNF (10 ng/ml, Peprotech), GDNF (10 ng/ml, Peprotech) and IGF1 (10 ng/ml, Peprotech) until day 24.

### Immunoﬂuorescence analyses

For immunocytochemistry, cells were ﬁxed with 4% paraformaldehyde for 20 minutes at RT, washed with PBS, and blocked with 10% bovine serum (Vector Laboratories) and 0.1% Triton X-100 (Sigma). Primary antibodies included β-III-TUBULIN (1:500, Sigma Aldrich), PCDH19 (1:100, Sigma), ZO-1 (1:100, Zymed.) Secondary antibodies were conjugated with Alexa 488, Alexa 555 or AlexaCy5 (Life Technologies). Coverslips were mounted using PBS/Glycerol (1:1), visualized using a confocal microscope Fluoview FV1000 (Olympus) and acquired with the software FV10-ASW Version 2.0.

### Confocal microscopy

Confocal microscopy was performed on a Leica TCS-SP8X laser-scanning confocal microscope (Leica Microsystems, Mannheim, Germany) equipped with white light laser (WLL) source, 405nm diode laser, SuperZ Galvo stage, 3 Internal Spectral Detector Channels (PMT) and 2 Internal Spectral Detector Channels (HyD) GaAsP. Sequential confocal images were acquired using a HC PLAPO 63x oil immersion objective (1.40 numerical aperture, Leica Microsystems) with a 1024×1024 format, scan speed 600Hz, an electronic zoom at 1.3 corresponding to 136 nm/pixel, and z-step size of 0.3 mm. Z-reconstructions (60 stacks) were imported into LAS AF 3D Analysis (Leica Microsystems) software to obtain their three-dimensional (3D) surface rendering.

## SUPPLEMENTARY MATERIAL FIGURES


