# Supplementary material for: Characterizing PCDH19 in human induced pluripotent stem cells (iPSCs) and iPSC-derived developing neurons: emerging role of a protein involved in controlling polarity during neurogenesis
Source: Oncotarget. 2015 Sep 21;6(29):26804–13. doi: 10.18632/oncotarget.5757 (PMC4694954; doi:10.18632/oncotarget.5757)
Supplement: Supplementary file 1 [file oncotarget-06-26804-s001.pdf]

Characterizing PCDH19 in human induced pluripotent stem cells (iPSCs) and iPSC-derived developing neurons: emerging role of a protein involved in controlling polarity during neurogenesis

Supplementary Material

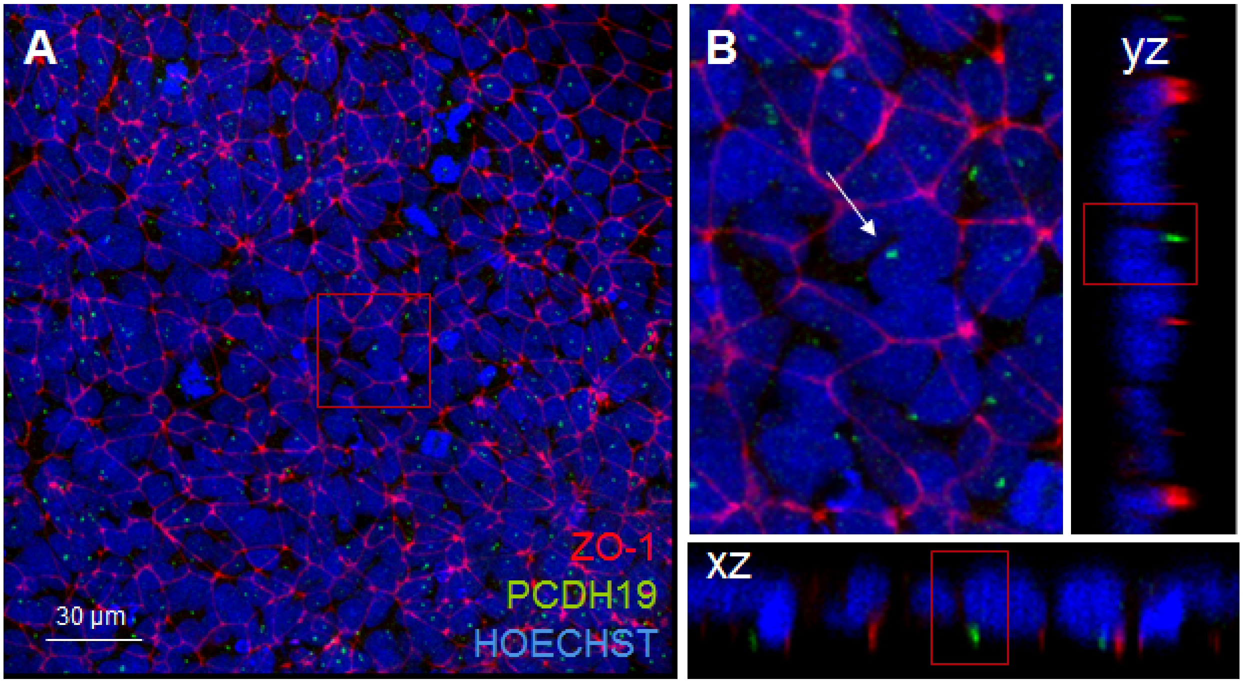

Supplemental Figure S1. PCDH19 localization in relation to ZO1 signal in proliferating iPSCs. Confocal photographs of iPSCs following immunofluorescence for PCDH19 (green) and ZO-1 (red) showing that the PCDH19 signal is limited to focal extra-nuclear foci. This localization is particularly evident in B, which is a higher magnification of the red box in A, with the xz and yz axes indicated on the sides.

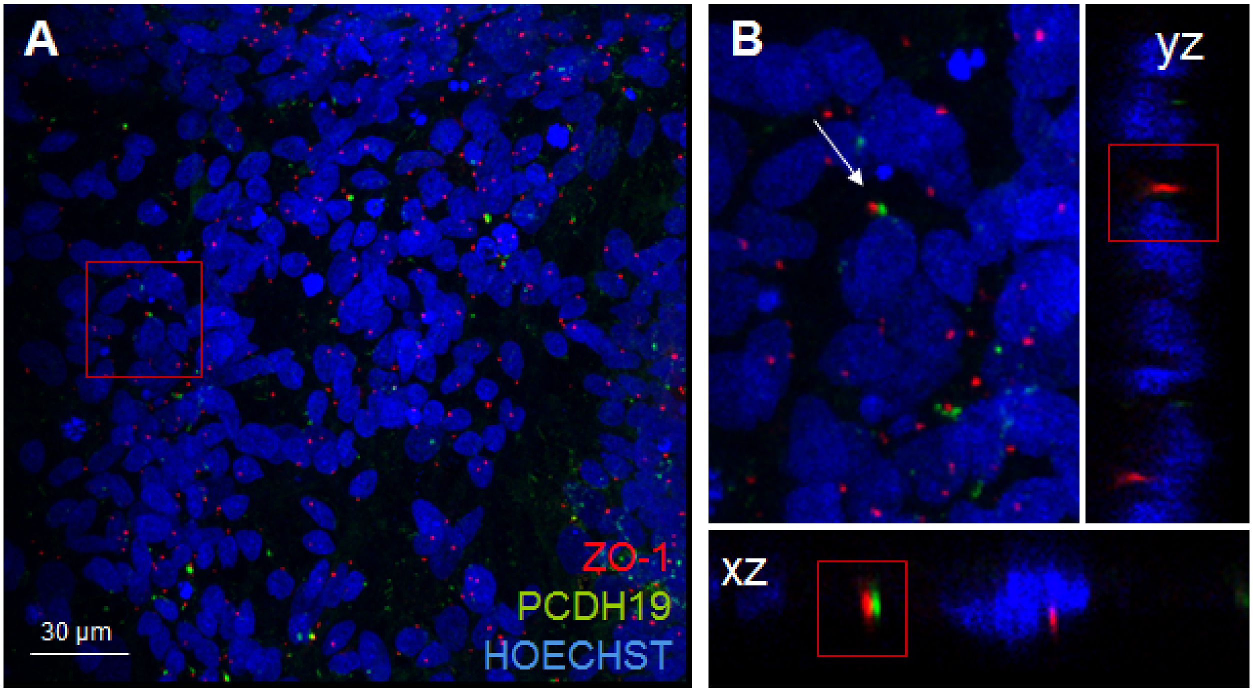

Supplemental Figure S2. PCDH19 localization in relation to ZO1 signal in iPSC-derived neurons. Immunofluorescence images for PCDH19 (green) and ZO-1 (red) (in A, B) of iPSC-derived neurons (after 30 days of differentiation) show that their signals are tighly juxtaposed but they do not co-localize, indicating that they both contribute to the definition of the cell-cell contacts. The red box in A indicates the region magnified in B with the xz and yz axes reported on the sides. This localization is particularly evident in B, which is a higher magnification of the red box in A, with the xz and yz axes indicated on the sides.
